# Clinical significance of tumor necrosis factor-alpha and carcinoembryonic antigen in gastric cancer

**DOI:** 10.25122/jml-2020-0098

**Published:** 2022-01

**Authors:** Mihai Cătălin Roșu, Petrut Dinu Mihnea, Andrei Ardelean, Silviu Daniel Moldovan, Romana Olivia Popețiu, Bogdan Dan Totolici

**Affiliations:** 1. Department of General Surgery, Faculty of Medicine, Vasile Goldiș Western University of Arad, Arad, Romania; 2. General Surgery and Clinical Laboratory, Emergency University Hospital Arad, Arad, Romania; 3. Department of General Surgery, Giurgiu County Emergency Hospital, Giurgiu, Romania

**Keywords:** tumor necrosis factor, carcinoembryonic antigen, gastric cancer, TNF alfa

## Abstract

Tumour necrosis factor (TNF)-α plays an important role in inflammatory, infectious, and tumor processes, and it is closely related to the early stages of gastric cancer. It is a pro-inflammatory cytokine, produced not only by macrophages and monocytes but also by the lymphocytes, mast cells, neutrophils, keratinocytes, smooth muscle cells, and some tumor cell lines. Large amounts of TNF are released upon contact of macrophages, CD4 + T lymphocytes, and natural killer (NK) cells with lipopolysaccharides, bacterial products, and interleukin 1. TNF-alpha inducing protein (Tipa) is a unique carcinogenic factor of Helicobacter pylori, secreted into culture broth. The biological activities of TNF alpha and deletion mutant were studied. TNF alpha protein specifically binds to cell-surface nucleolin and then enters the gastric cancer cells, where TNF-a and chemokine gene expressions are induced by NF-jB activation. Nucleolin localizes on the surface of gastric cancer cells, and interaction between TNF alpha and cell-surface nucleolin causes a cancer-oriented microenvironment that increases the risk of gastric cancer. This paper discusses a mechanism of gastric cancer development and the clinical significance of tumor necrosis-alpha and carcinoembryonic antigen in gastric cancer.

## Introduction

The carcinoembryonic antigen (CEA) is a glycoprotein with a molecular weight of 200 kDa, containing approximately 40% proteins and 60% carbohydrates. CEA is one of the oncofetal antigens produced during embryonic life and fetal development. From the immunohistological perspective, CEA may be determined in high concentrations in the fetal gastrointestinal tract and pancreas, colorectal adenocarcinomas, and low concentrations in normal tissue and the intestinal mucosa, exocrine pancreas, and liver [1, 2]. Around 990,000 people are diagnosed with gastric cancer (GC) every year, out of which about 738,000 die. Gastric cancer is the fourth most common cancer and the second most common cause of cancer death [1]. The incidence of gastric cancer depends on gender and geographic variation. Men are 2–3 times more sensitive than females. Incidence shows a great deal of geographical diversity. It should be noted that more than 50% of new incidents occur in developing countries [1]. The incidence of gastric cancer has declined in most parts of the world over the past few decades. Sporadic intestinal gastric cancer type declines have been observed, and the incidence of diffuse GC types has increased. Proximal GC velocities are higher than distal GC velocities [3–17]. The rapid progression of gastric cancer, with no specific clinical symptoms, makes this disease a challenge in terms of diagnosis and treatment in its early stages, which may contribute to a higher mortality rate. Surgical resection remains the major treatment method, although the prognosis for patients with unresected gastric cancer is poor [3–5]. Up to the present, we know about several risk factors such as dietary factors, infection with Helicobacter Pylori bacteria, and Epstein-Barr virus [2]. At the same time, more and more studies highlight genetic factors and their influence on cellular processes, which play an important role in developing this cancer and the disease mechanism [2]. Despite the results of the diagnosis and treatment of gastric cancer, the prognosis of these patients remains poor, with 5-year survival, which is why identifying and correlating these specific biologics markers are important and effective in the diagnosis and treatment of patients with gastric cancer [6–8].

Serological tumor markers are widely used for the diagnosis, the effect of the treatment, and gastric cancer surveillance. Numerous studies have assessed and analyzed the plasma values of tumor markers for gastric cancer, but no correlation has been made between TNF alpha, the carcinoembryonic antigen, CA19-9, and CA 72-4 [9–11]. A study conducted on 587 patients diagnosed with gastric cancer analyzed the CA 19-9, the alpha-fetoprotein, and the CA 125 biomarkers from 2008 until 2015 as biomarkers, and all patients had proximal, distal, or total gastrectomy performed at the D2 level. Data such as age, sex, tumor location, and serum values of the biomarkers listed above were included [16]. A close correlation between the elevated tumor markers and the clinicopathological manifestations in gastric cancer was highlighted. The researchers concluded that the increased CA 19-9 levels might be associated with gastric cancer developed in the female patients and the presence of metastatic lymph nodes. Carcinoembryonic antigen (CEA) levels have been associated with unfavorable progression in newly diagnosed gastric cancer [12–15]. CEA may be correlated with tumor thickness and liver metastases; it emerged in the pancreas and the gastrointestinal tract as a cell surface antigen. However, other studies have shown that CA 19-9 was correlated with tumor staging.

Inflammation is considered the most important factor in the pathogenesis of cancer, so this is why the polymorphism of genes involved in inflammation has been carefully studied in recent years. TNF alpha is the most studied marker in gastric cancer, and it has been shown that at the cellular level, its polymorphism is of particular importance as the gene is identified at the level of chromosome 6. The biologically active form of TNF-α is the trimer, and, in addition to this soluble form, there is also a membrane-bound form located on the surface of the TNF alpha-producing cells, which serves as a support for the soluble form by proteolytic cleavage. The aim of this review paper is to highlight the clinical significance of these specific markers, i.e., TNF alpha and CEA, for the development of gastric cancer as well as for the risk of other inflammatory conditions and a possible correlation of other biomarkers like CA 72-4 in patients with gastric cancer.

## Material and Methods

We searched the PubMed database for studies related to the 2 proteins (i.e., TNF alfa and CEA, respectively) we have chosen in the study. Consequently, we selected the most recent and significant results to illustrate the clinical significance of the two markers and their correlations in patients diagnosed with gastric cancer and intestinal inflammation. We studied articles from the database from 2008 to 2020, respectively 28 articles about TNF alpha and 16 CEA articles related to gastric cancer.

## Results

Recent studies show that serum concentrations of TNF alpha and CEA may be associated with gastric cancer and the pathogenesis of the gastrointestinal disease. TNF alpha and CEA may be potential predictors of gastric cancer in patients with benign gastric lesions. Serum CEA levels provide additional prognostic information for patients with primary gastric cancer. In particular, elevated serum CEA levels provide additional prognostic information and are useful predictors of curability in patients undergoing gastrectomy. Serum CEA levels are an independent prognostic factor in patients with primary gastric cancer [3, 5–8].

TNF-alpha clearly plays a major role in establishing a link between inflammation and cancer. Because TNF-alpha is also needed for the proper functioning of the immune system, complete suppression of TNF-alpha over a long period is likely to prove harmful. Any chronic inflammatory condition linked to the majority of the inflammatory diseases could be a potential target for anti-TNF-alpha therapy [11–17].

## Discussion

The tumor necrosis factor TNFαlpha plays an important role in the process of inflammation, infection, and tumors and has central importance in the early stages of gastric cancer. The relationship between TNFα and CEA is well established but contradictory and controversial. A pro-inflammatory cytokine genetic profile increases the risk of non-cardia gastric adenocarcinoma but not other upper gastrointestinal cancers, possibly by inducing a hypochlorhydria and atrophic response to gastric H. pylori infection.

## Conclusions

This review described the characteristics of gastric cancer, taking into account epidemiology, risk factors, and possible correlations between TNFα and CEA. Geographical variations and gender need to be taken into account. Several risk factors such as family history, diet, alcohol, smoking, Helicobacter pylori, and Epstein-Barr infection are also important. Polymorphisms in the TNF-α promoter affect GC risk. High TNF producer alleles are associated with increased risk. The TNF-α polymorphism is H. It may enhance carcinogenesis through chronic inflammation of Helicobacter pylori. Previous studies have outlined many controversial results based on the correlation between specific and non-specific biomarkers of gastric cancer. Therefore, there is an urgent need to identify other links between biomarkers, immunohistochemical studies, and molecular biology tests.

## Acknowledgments

### Conflict of interest

The authors declare no conflict of interest.

### Authorship

MCR and BDT designed and conducted the research. SDM and ROP revised the manuscript and made critical changes. PDM and AA collected and analyzed data to reduce bias.
